# Optimization of Genome Engineering Approaches with the CRISPR/Cas9 System

**DOI:** 10.1371/journal.pone.0105779

**Published:** 2014-08-28

**Authors:** Kai Li, Gang Wang, Troels Andersen, Pingzhu Zhou, William T. Pu

**Affiliations:** 1 Deparment of Cardiology, Boston Children’s Hospital, Boston, MA, United States of America; 2 University of Copenhagen, Copenhagen, Denmark; 3 Harvard Stem Cell Institute, Harvard University, Cambridge, MA, United States of America; Michigan State University, United States of America

## Abstract

Designer nucleases such as TALENS and Cas9 have opened new opportunities to scarlessly edit the mammalian genome. Here we explored several parameters that influence Cas9-mediated scarless genome editing efficiency in murine embryonic stem cells. Optimization of transfection conditions and enriching for transfected cells are critical for efficiently recovering modified clones. Paired gRNAs and wild-type Cas9 efficiently create programmed deletions, which facilitate identification of targeted clones, while paired gRNAs and the Cas9D10A nickase generated smaller targeted indels with lower chance of off-target mutagenesis. Genome editing is also useful for programmed introduction of exogenous DNA sequences at a target locus. Increasing the length of the homology arms of the homology-directed repair template strongly enhanced targeting efficiency, while increasing the length of the DNA insert reduced it. Together our data provide guidance on optimal design of scarless gene knockout, modification, or knock-in experiments using Cas9 nuclease.

## Introduction

Designer site-specific nucleases such as TALENs [1], [2] and CRISPR/Cas9 [3], [4] have opened the door for rapid genome engineering. By introducing targeted double strand breaks into a genome, these nucleases stimulate endogenous repair mechanisms. One repair mechanism is non-homologous end joining (NHEJ), in which a double strand break is repaired by joining the DNA ends. NHEJ repair is error-prone and causes insertions and deletions (indels) around the DNA break point. In the presence of a donor DNA template with homology to the region of the DNA break, homology-directed repair (HDR) occurs. By placing sequence variations or exogenous DNA fragments between homology arms, the HDR mechanism can be leveraged to program specific insertions, deletions, or base changes at a target locus.

With the aid of designer site-specific nucleases, targeted genome modification can be achieved with sufficient efficiency that the desired modifications can be recovered without use of positive selection cassettes [3]–[5]. This is an important advance over more traditional gene targeting strategies, since it obviates the need to remove the selection cassette. Selection cassette removal requires additional handling and moreover typically leaves residual sequences. When first described using the CRISPR/Cas9 system, “scarless” genome editing efficiency was in the 1–4% range [4], [5]. At this efficiency, a large number of clones need to be screened to obtain a single targeted clone. Screening strategies involving selective amplification of cell pools containing targeted clones have been described that overcome this limitation [6]. However, increasing the frequency of targeted clone recovery represents an alternative and more direct approach to optimize scarless genome editing.

Here, we report a series of experiments that investigate several parameters that influence the efficiency of scarless genome editing using the CRISPR/Cas9 system. Key parameters include (a) optimization of transfection efficiency; (b) enrichment for transfected cells; (c) integration of downstream genotyping into the design of the targeting strategy; and (d) optimizing the design of the donor DNA template. Through these improvements, we increased the efficiency of genome editing: we routinely achieved 30–60% efficiency for homozygous targeted gene knockout through NHEJ, and 4% efficiency for addition of a GFP epitope tag onto endogenous genes.

## Materials and Methods

### Plasmids and oligonucleotides

The pCas-9-GFP, pCas9D10A-GFP, and gRNA cloning vectors were obtained from Addgene. The traditional MEF2C FlagBio target vector containing the FlagBio ORF and Frt-Neo-Frt cassette flanked by 1 kb and 4 kb homolog arms was made by recombineering [7]. The dsDNA donor with 50 bp homolog arms on each side was made by PCR, using primers harboring 50 bp homology sequences. The *Oct4*-GFP donor with 200 bp homolog arms was made using the Gibson assembly technique [8] using a kit from New England Biolabs, with pCR-Blunt II-TOPO vector (Life Technologies) as backbone.

Oligonucleotides were synthesized by Integrated DNA Technologies. Primer sequences are indicated in Table 1. gRNAs were designed using the CRISPR design tool (crispr.mit.edu) [9].

**Table 1:** Oligonucleotides used in this study.

qPCR primers:	
MLL2-qPCR-F	tgttcccggctttaccacttt
MLL2-qPCR-R	ggctccatgatagtgatgccc
CHD7-qPCR-F	gacccagggatgatgagtctt
CHD7-qPCR-R	atggggttcacggggttttc
GAPDH-qPCR-F	acagtccatgccatcactgcc
GAPDH-qPCR-R	gcctgcttcaccaccttcttg
**gRNAs:**	
GFP KO gRNA #3	ggccacaagttcagcgtgtc cgg
GFP KO gRNA #4	ggcgagggcgatgccaccta cgg
GFP KO gRNA #5	ccggcaagctgcccgtgccc tgg
GFP KO gRNA #9	cttcagggtcagcttgccgt agg
SMAD2 KO gRNA #3	gccgtcttcaggtttcacac cgg
SMAD2 KO gRNA #4	tcacagtcatcatgagctca agg
CHD7 KO gRNA #1	ccagggatgatgagtctttt tgg
CHD7 KO gRNA #2	gaaaaccccgtgaaccccat ggg
MLL2 KO gRNA #1	tccgaaacatgtaaataccg cgg
MLL2 KO gRNA #2	ctgtgcccctaactgtgtag cgg
sap130 gRNA #2	catgtccaacacttaccaac tgg
**primers for genotyping:**	
smad2-F	ggctctccttcatgtcctctt
smad2-R	ccagcctttagtcgtcctctt
CHD7-F	ccactcctttccaggaccta
CHD7-R	tcattcggttaggctgatcc
MLL2-F	tttgaatccaggaggcagtt
MLL2-R	gtcaaggcaggcctgatg
sap130-F	ctgtgtcgcacacacaagc
sap130-R	cctagggcttactggccaac
**SAP130-DONOR**	tcatcccatgctactgctgtgaccacttcaaatatcccagttggcaagtgttggacatgtcgcacaaaagcctttgaggaatttgcaggc
**50 bp-homolog FlagBio donor for Mef2c:**	
mef2c-flagbio-F	gggaaagtccttcagtcaagcgcatgcgactctctgaaggatgggcaacagactacaaagacgatgacga
mef2c-flagbio-R	gcacacacacacactgcaagaaaaaaaaaaactattaagtaataatgtgatcacctcgagctcggcgcgc
**50 bp-homolog GFP donor for Mef2c:**	
mef2c-gfp-F	gggaaagtccttcagtcaagcgcatgcgactctctgaaggatgggcaacagtgagcaagggcgaggagct
mef2c-gfp-R	gcacacacacacactgcaagaaaaaaaaaaactattaagtaataatgtgattacttgtacagctcgtcca
**50 bp-homolog GFP donor for OCT4:**	
OCT4-GFP-F	cctttccctctgttcccgtcactgctctgggctctcccatgcattcaaacgtgagcaagggcgaggagct
OCT4-GFP-R	gtctacctcccttgccttggctcacagcatccccagggagggctggtgccttacttgtacagctcgtcca
**Gibson assembly to make 200-bp homolog donor for OCT4:**	
200****bp_right-F	ttggtaccgagctcgcccaacgagaagagtatg
200****bp_right-R	ttgctcacgtttgaatgcatgggagag
GFP-F	cattcaaacgtgagcaagggcgaggag
GFP-R	ctggtgccttacttgtacagctcgtccatg
200****bp_left-F	gtacaagtaaggcaccagccctccctgg
200****bp_left-R	ggcgaattgggcccttatttaagaacaaaatgatgagtgacagacaggcc

### Cell Culture

Mouse J1 ESCs (ATCC) were cultured on MEF-coated gelatinized plates at 37 in 5% CO_2_ in ES medium. ES medium contained Dulbecco’s modified Eagle’s medium (DMEM) (high glucose, Life Techologies) supplemented with 15% ES-qualified fetal bovine serum (Invitrogen), LIF (Millipore), GlutaMAX, 2-mercaptoethanol, MEM non-essential amino acids, and penicillin/streptomycin.

### Transfection

Traditional electroporation was done using a Gene Pulser electroporator (BioRad) at 250 V, 500 uF. Nucleofection was performed using the mouse ESC nucleofection kit (VPH-1001; Amaxa). Lipofectamine 3000 (Life Technologies, L3000008) and Xfect (Clontech) were used according to manufactures’ protocols. To enrich for transfected cells, GFP FACS was performed 48 hours after transfection. Alternatively, pgk-puromycin plasmid was co-transfected with Cas9 in a 2∶3 ratio. After 48 hours, cells were selected in 1.5 µg/ml puromycin for 48 hours.

### Genotyping and surveyor assay

Mouse ESCs were digested overnight in lysis buffer (10 mM Tris-Hcl 7.5, 10 mM EDTA, 10 mM NaCl, 1% SDS, and 1 µg/ml proteinase K). Genomic DNA was purified by ethanol precipitation. PCR primers flanking the target regions were designed by Primer 3 software. For Surveyor nuclease assay, 800 ng PCR products were melted and annealed in 1X Taq buffer for heteroduplex formation. The hybrid DNA was treated with 1 µl enhancer S and 1 µl nuclease S according to manufacture’s protocol. The reaction products were analyzed on 4–20% Novex TBE polyacrylamide gels (Life Technologies), and visualized by Sybr Gold nucleic acid staining (Life Technologies, S-11494).

### Gene Expression

RNA was purified using the RNeasy mini kit (Qiagen, 74104). cDNA synthesis was performed using Superscript III first-strand synthesis system for RT-PCR (Life Technologies, 18080-051). Quantitatative PCR was performed with Sybr Green chemistry, using GAPDH as the endogenous control. Primer sequences are indicated in Table 1.

## Results

**Figure 1:**
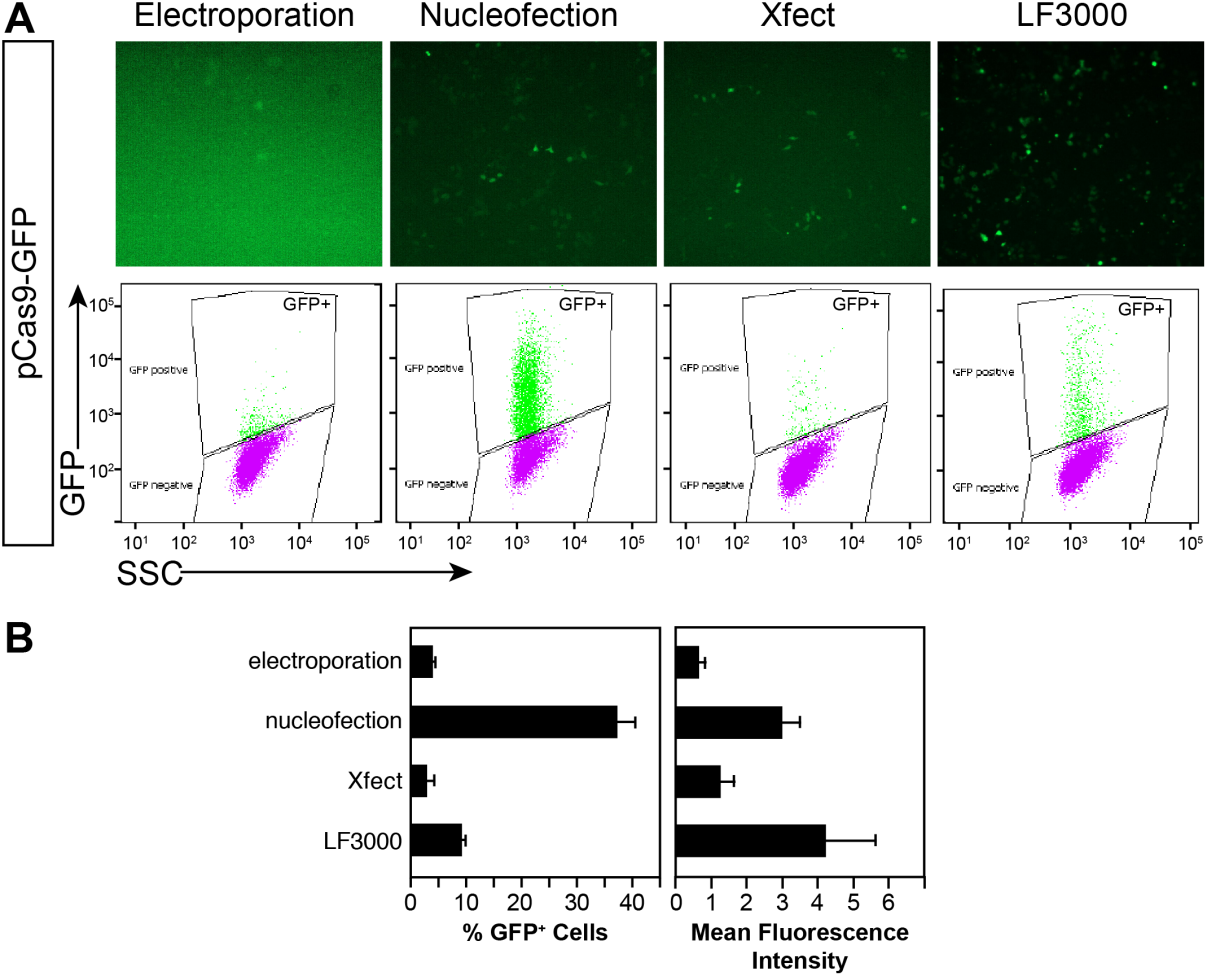
Optimization of mESC transfection. **A.** pCas9-GFP expression plasmid was transfected into mESCs by the indicated method. GFP fluorescence was assessed by fluorescent microscopy and FACS. Numbers indicate the fraction of GFP-expressing cells. **B.** Percent of transfected cells and mean fluorescence intensity of GFP+ cells. n = 3. Graphs show mean ± s.e.m.

The CRISPR system offers unprecedented genome editing capability. Ideally, such genome-editing would be scarless (i.e. without need for a selection cassette) yet highly efficient. A major factor that reduces the fraction of genome-edited clones in the absence of positive selectable markers incorporated into the modified allele is transfection efficiency. To deliver CRISPR components more efficiently into mouse ES cells (mESCs), we tested four different transfection methods: traditional electroporation; nucleofection; and two different, recently introduced liposomal agents (Xfect and Lipofectamine 3000 (LF3000)). We transfected Cas9-GFP expression plasmids to mESCs, and analyzed the percentage of GFP positive cells by fluorescence-activated cell sorting (FACS; Figure 1). Nucleofection and LF3000 performed the best or second best in three replicate experiments that measured the fraction of transfected cells and the mean fluorescence intensity (Figure 1B). In subsequent experiments, we used either nucleofection or LF3000 since these gave the highest transfection efficiency for the pCas9-GFP plasmid, the active reagent in these genome editing experiments.

### Optimization of CRISPR-mediated gene knockout

**Figure 2:**
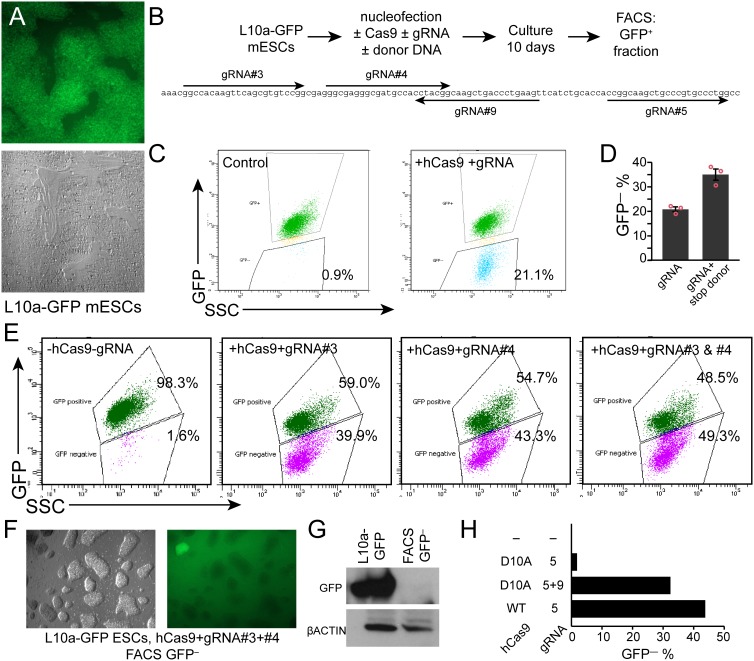
Cas9 gene knockout using single or dual gRNAs. **A.** mESC cell line L10a-GFP, in which one Rosa26 locus expresses GFP. **B.** Experimental outline and gRNAs. gRNAs #9 and #5 generate 5′ overhangs for dual nickase strategy. **C.** Single gRNA-directed GFP inactivation. **D.** Comparison of gRNA alone or gRNA+HDR donor containing a translational stop signal. n = 3. Bar = s.e.m. Red circles indicate individual data points. **E.** Gene inactivation frequency of single compared to paired gRNAs. **F–G.** Assessment of GFP expression in mESCs in the GFP-FACS fraction by fluorescent microscopy and western blotting. **H.** Effective gene knockout using dual gRNAs and the hCas9-D10A nickase mutant.

We established a system in which we could easily monitor the efficiency of CRISPR gene knockout. Our lab previously described a mESC line in which a CAG-promoter driven L10a-GFP fusion protein was knocked into the Rosa26 locus [10]. These heterozygous L10a-GFP mESCs robustly express GFP (Fig. 2A). We co-transfected these cells with hCas9 and a gRNA directed against GFP (Fig. 2B). Subsequent FACS analysis showed that 21.1% of cells were GFP^−^ after transfection (Fig. 2C), compared to 0.9% in cells not treated with hCas9.

Expression of hCas9 and a target-specific gRNA causes gene knockout by introducing a double strand break, which is repaired by non-homologous end joining (NHEJ). In the presence of the appropriate DNA donor, the double strand break also stimulates homology-directed repair (HDR). We hypothesized that programming a DNA donor template with a stop codon would increase the efficiency of generating knockouts through the HDR pathway. We tested this hypothesis on the L10a-GFP locus. We found that hCas9+gRNA alone (NHEJ) yielded 20.7±0.9% GFP^–^ cells, while hCas9+gRNA+DNA donor (both NHEJ and HR) yielded 34.9±2.3% GFP^−^ cells (P<0.05; Fig. 2D). Although the knockout efficiency varied based on the transfection efficiency, the rank order was consistent between trials (Fig. 2D). Thus, adding a donor oligo with stop codon increased KO efficiency compared to hCas9 plus gRNA alone.

Next, we considered whether the number of gRNAs influenced Cas9 knockout efficiency. We compared GFP knockout in L10a-GFP mESCs treated with one gRNA (#3 or #4) or both. hCas9 was co-transfected with gRNA #3, gRNA #4, or both into L10a-GFP mESCs. We observed 39.9% GFP^–^ cells using gRNA #3, 43.3% GFP^–^ cells using gRNA #4, and 49.3% using both gRNAs (Fig. 2E). We confirmed GFP knockout by microscopic imaging of replated, FACS-sorted GFP^–^ cells (Fig. 2F). Furthermore, western blotting confirmed loss of GFP protein expression in the sorted GFP^–^ cells (Fig. 2G). We genotyped GFP-clones targeted by two gRNAs by Sanger sequencing of PCR amplicons and confirmed deletion between two gRNA cut sites. Since dual gRNAs do not impair and may enhance knockout efficiency, and since paired gRNAs induces a deletion that facilitates genotyping (see below), in the following experiments we typically used two gRNAs per target gene to simplify detection of genome modification.

The frequency of off-target Cas9 activity can be reduced using a Cas9 point mutant, hCas9-D10A, which introduces single nicks rather than double strand breaks [11]–[13]. We tested the effectiveness of the hCas9-D10A nickase for generating gene knockouts (Fig. 2H). When we co-transfected hCas9-D10A nickase with one gRNA #5, the efficiency of ablating GFP was low (1.72%), as expected since DNA nicking should not induce NHEJ-mediated mutagenesis. The same individual gRNA co-transfected in parallel with wild-type hCas9 yielded 43.7% GFP^–^ cells. However, hCas9-D10A co-transfected with a second gRNA designed to introduce a nick on the opposite strand with 5′ overhangs (gRNA #5 and gRNA #9, Fig. 2B) [11] yielded 32.3% GFP^–^ cells. While this efficiency is slightly lower than that achieved using the wild-type hCas9, this reduction is may be a worthwhile tradeoff for reduced off-target mutagenesis with hCas9-D10A.

**Figure 3:**
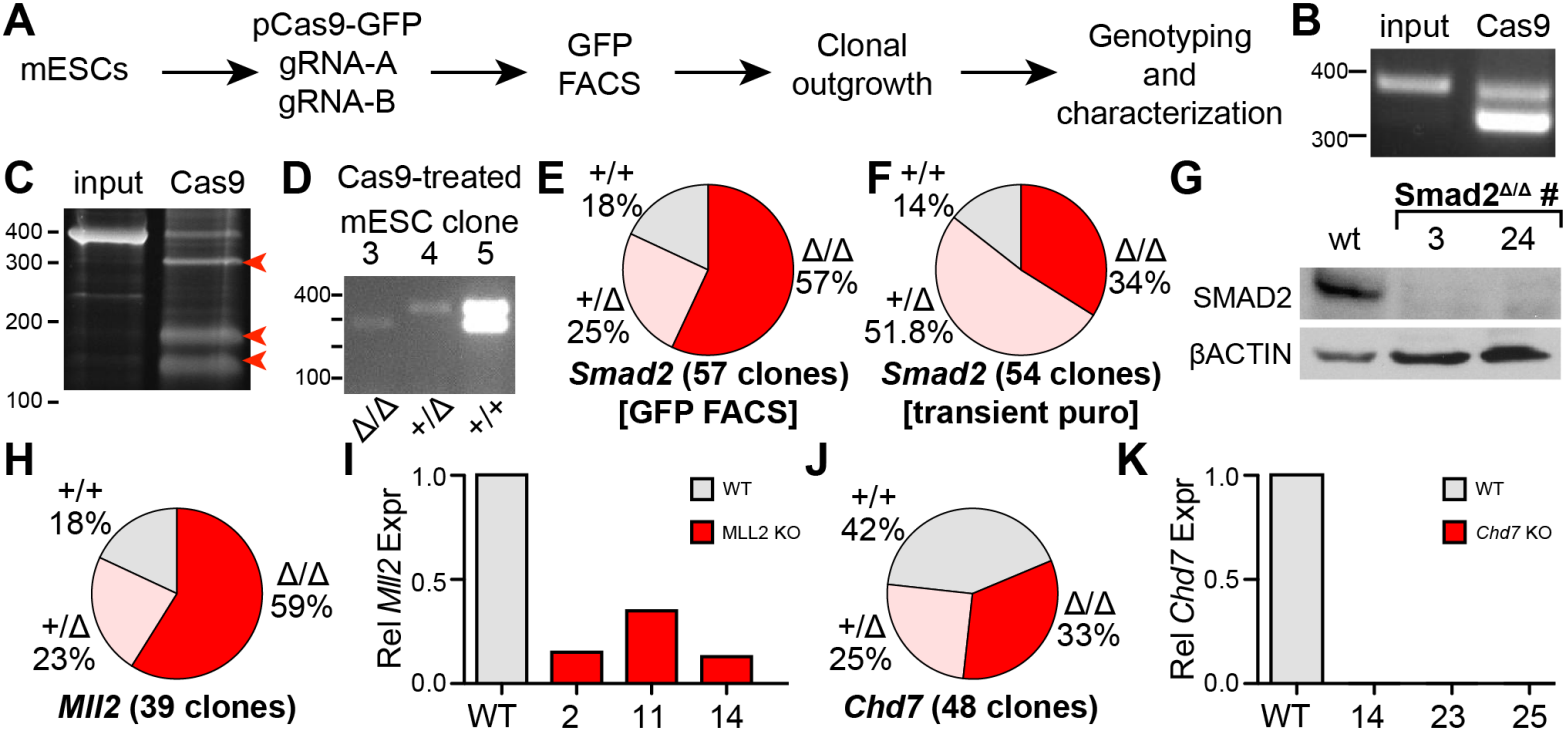
Knockout of 3 congenital heart disease genes in mESCs. **A.** Experimental outline. Paired gRNAs are designed to induce a deletion of about 100 bp. **B.** PCR genotyping of pooled ESC genomic DNA after *Smad2* targeting. The mutant PCR product predominated. **C.** Surveyor nuclease on pooled ESC genomic DNA. Red arrowheads indicate nuclease cleavage products indicative of heterodimers. **D.** PCR genotyping of individual clones showing Smad2^Δ/Δ^, Smad2^+/+^, and Smad2^+/Δ^ clones. **E–F.** Frequency of *Smad2* genotypes amongst clonal outgrowths, after enrichment for transfected cells by GFP FACS or by transient puromycin selection. **G.** Western blot measurement of *Smad2* expression in wild-type and Smad2^Δ/Δ^ clones. **H.** Frequency of *Mll2* genotypes amongst 39 genotyped clones. **I.** Confirmation of *Mll2* inactivation in individual Mll2^Δ/Δ^ clones by qRTPCR. **J.** Frequency of *Chd7* genotypes amongst 48 genotyped clones. **K.** Confirmation of *Chd7* inactivation in individual Chd7^Δ/Δ^ clones by qRTPCR.

Having defined parameters that permit efficient Cas9-mediated gene knockout in mESCs, we next targeted Smad2, MLL2, and CHD7, mutations of which have been implicated in human congenital heart disease [14] (Fig. 3A). First, we targeted Smad2 in NKX2.5-GFP mESCs, in which the cardiomyocyte-specific NKX2.5 promoter drives GFP expression, by co-transfecting pCas9-GFP with two gRNAs for Smad2. We performed GFP FACS to enrich for cells with high Cas9-GFP expression and then clonally expanded them. We analyzed genomic DNA from clones to determine the Smad2 knockout efficiency. PCR amplification of pooled genomic DNA using primers flanking both gRNA-target sites showed that hCas9 plus two gRNAs generated the expected full length PCR product plus a smaller product, which corresponded to mutant alleles containing a deletion. Notably, the smaller PCR product was more intense than the wild-type band, suggesting highly efficient, targeted mutagenesis (Fig. 3B). We confirmed this result by treating PCR-amplified pooled genomic DNA with surveyor nuclease, which cleaves heteroduplex DNA, on pooled genomic DNA. Compared to untreated cells, Cas9 with gRNA treated cells yielded additional nuclease cleavage products (Fig. 3C). PCR genotyping showed that 32 out of 56 clones (57%) were homozygous mutants, 14 out of 56 (25%) were heterozygous mutants, and 10 out of 56 (18%) were wild-type (Fig. 3D–E). This likely underestimates the mutagenesis efficiency, since the larger PCR band may actually contain smaller indel mutations, which could occur if only one of the two gRNAs cleaved its target.

In scarless genome editing without positive selection, enrichment for transfected cells is an important step to increase the fraction of colonies that harbor a mutation of interest. We compared targeted *Smad2* mutagenesis efficiency using FACS-selection of GFP^+^ cells transfected with Cas9-GFP (above) to an alternative procedure in which Cas9 expression plasmid was co-transfected with pgk-puro. 48 hours after transfection, cells underwent 48 hours of puromycin selection. We genotyped 54 independent clonal outgrowths and obtained 19 (34%) homozygous mutants, 29 (51.8%) heterozygous mutants, and 8 (14.3%) without apparent modification (Figure 3F). These data indicate that co-transfection with pgk-puro and transient puromycin selection or co-expression of GFP with FACS enrichment are both suitable for enrichment of transfected cells in Cas9 genome editing experiments, although FACS selection for GFP may yield a greater proportion of targeting of both alleles.

We sequenced 9 clones in which both *Smad2* alleles were mutated based on the PCR genotyping assay. The Sanger sequencing data showed that all of these clones did indeed have homozygous deletion of the region between the two gRNAs. We further confirmed *Smad2* knockout at the protein level by performing western blotting. SMAD2 protein was indeed ablated in the clones assigned the Smad2^−/−^ genotype by the PCR genotyping assay (Fig. 3G).

We used the same knockout strategy with GFP FACS enrichment to mutate *Mll2* and *Chd7* in mESCs. PCR genotyping demonstrated that mESCs targeted with hCas9-GFP and two gRNAs were efficiently mutated at the *Mll2* and *Chd7* loci: 23 of 39 clones (59%) genotyped contained substantial homozygous *Mll2* deletions (Fig. 3H–I), and 16 of 48 clones (33%) genotyped contained substantial homozygous *Chd7* deletions (Fig. 3J–K). We confirmed reduction of *Mll2* and *Chd7* mRNA expression in 3 independent clones of each target locus (Fig. 3I, K). Although these clones had confirmed gene deletions, only some had reduced transcript level, likely due to variability in nonsense-mediated decay.

These data demonstrate that the CRISPR system achieves highly efficient scarless genome editing at several different loci. Using optimized transfection conditions and FACS selection of transfected cells by co-transfected GFP fluorescence yields highly efficient mutagenesis. Use of two gRNAs permits efficient identification of mutated clones by PCR genotyping. The hCas9D10A mutant also achieves efficient mutagenesis with potentially reduced off-target activity.

### Targeted DNA base changes using CRISPR

**Figure 4:**
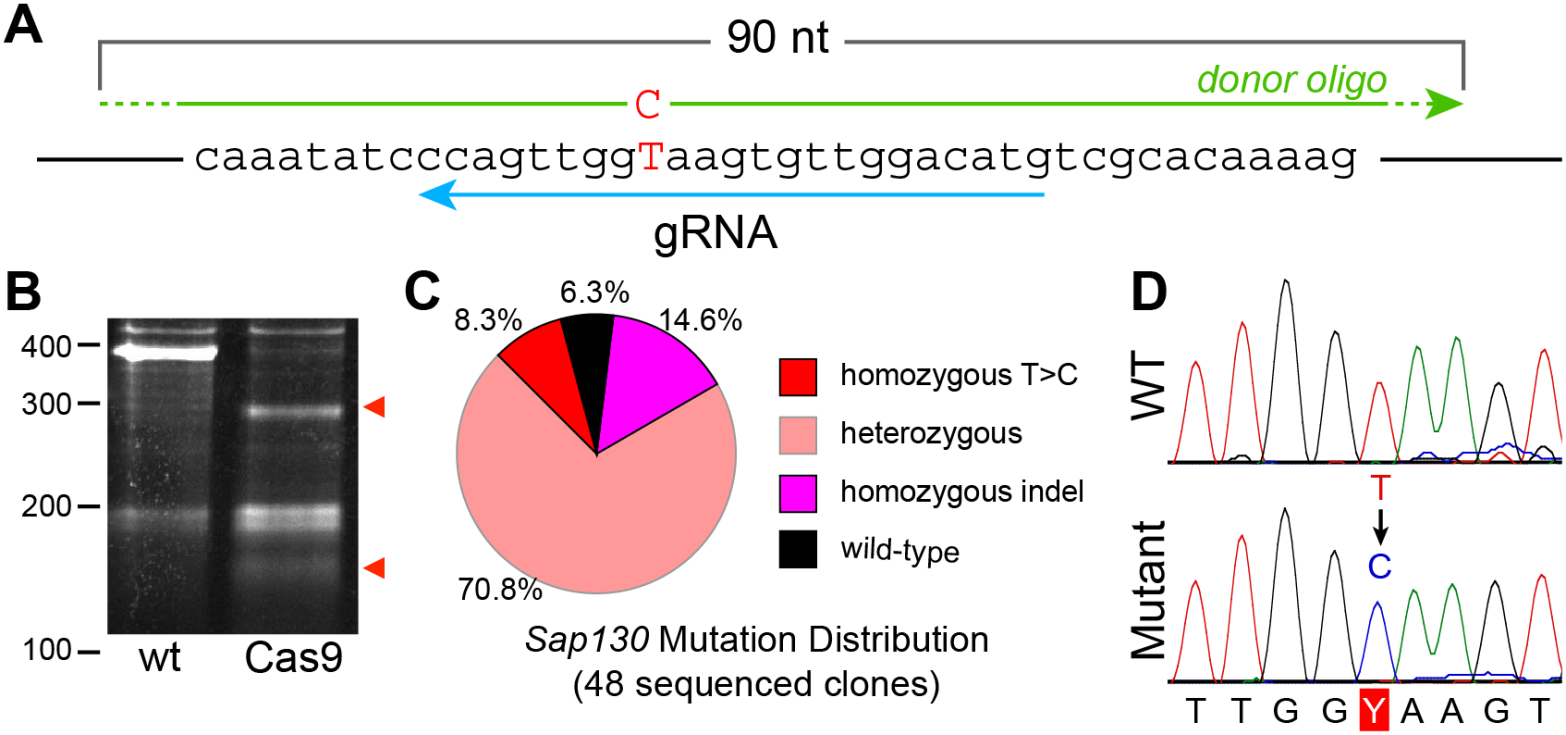
Cas9-mediated introduction of a single base change. **A.** *Sap130* targeting strategy. The blue arrow indicates the gRNA targeting the reverse strand with the tail and tip representing the 5′ and 3′ ends, respectively. The HDR donor oligo is indicated by the green arrows. The T>C change is indicated in red. **B.** Surveyor nuclease assay on pooled ESC genomic DNA. Red arrowheads indicate nuclease cleavage products, diagnostic of heterodimers. **C.** Distribution of *Sap130* mutations. **D.** Sequence chromatograms showing homozygous T>C mutation.

Having successfully used CRISPR to knock out genes in mouse ES cells, we further evaluated the utility of CRISPR for targeted gene modification, rather than simple knockout. We assessed the efficiency of Cas9 to introduce a T>C mutation into the gene *Sap130*. We used nucleofection to introduce pCas9-GFP, *Sap130*-targeted gRNA, and donor oligo harboring the desired T>C mutation into mESCs (Fig. 4A). We performed FACS sorting to select transfected, GFP-expressing cells, and then clonally expanded them. Conducting the surveyor nuclease assay on pooled genomic DNA demonstrated highly efficient genome modification, since nearly all of the PCR products were cleaved by the nuclease (Fig. 4B). PCR amplification of genomic DNA followed by Sanger sequencing for 48 clones showed that 4 clones (8.3%) were homozygous for the desired T>C mutation. Of the remaining clones, 34 (70.8%) contained different sequences at the two alleles (“heterozygous”), 3 (6.3%) were wild-type (Fig. 4C), and 7 (14.6%) had the same indel mutation on both alleles (“homozygous indel”). The frequency of homozygous indel mutants was surprising, and we confirmed this result for 2 randomly selected clones by cloning the genomic DNA PCR product and sequencing 16 independent clones. One potential reason for this observation is that the mutagenic event on one allele might be transferred to the second (e.g by inter-allelic gene conversion). Overall, our data indicate that scarless introduction of a targeted point mutation can be efficiently achieved using this approach.

### Targeted DNA insertions using CRISPR

**Figure 5:**
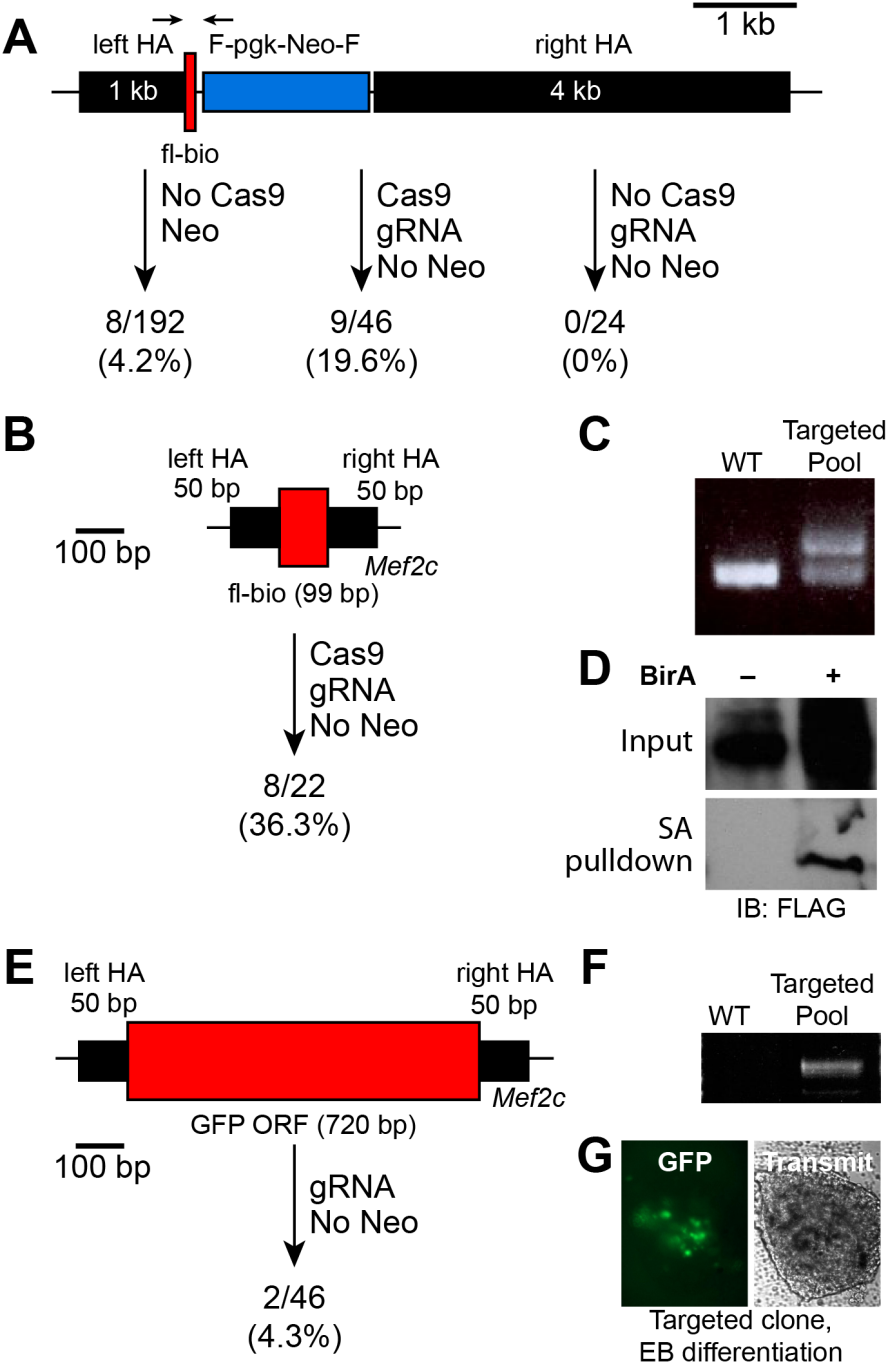
Cas9-mediated targeted knockin. **A.** Mef2c traditional targeting vector containing long homology arms, fl-bio epitope tag, and positive selection cassette. Efficiency of traditional and Cas9-mediated targeting strategies is shown. **B.** Cas9-mediated targeting using a 50 bp homology arms and no selectable marker. **C.** PCR genotyping of pooled ESC genomic DNA. Upper band is diagnostic of knockin. **D.** Confirmation of knockin and effective epitope tagging by streptavidin pulldown of in vivo biotinylated Mef2c. **E.** Cas9-mediated knockin of a longer insert. **F.** PCR genotyping of pooled ESC genomic DNA. The band is diagnostic of knockin. **G.** GFP expression after EB differentiation of a targeted clone.

We next evaluated the ability of CRISPR to introduce an exogenous DNA sequence, such as an epitope tag, into a target locus. We already had in hand a traditional targeting vector designed to introduce FLAG and BIO [15] epitope tags onto the C-terminus of *Mef2c*. This targeting vector contained 1 kb and 4 kb homology arms, the 99 bp FLAG-BIO tag, and a 1.8 kb *pgk*-Neo resistance cassette (Fig. 5A). In a traditional gene targeting experiment without Cas9 and with G418 selection, we obtained 8 correctly targeted clones out of 192 screened mESCs (4.2%). In comparison, we transfected the targeting vector with hCas9-GFP and appropriate gRNA. We then FACS selected the transfected, GFP^+^ cells, and clonally expanded them without G418 selection. Out of 46 clones genotyped, 9 were properly targeted (19.6%; Fig. 5A). In comparison, omission of Cas9 yielded no correct clones out of 24 tested (P = 0.023, Fisher’s exact test; Fig. 5A). These data show that CRISPR greatly increases HDR efficiency and facilitates knockin gene modification of foreign DNA sequences up to several kilobases in length.

Traditional targeting vectors have long homology regions and a positive selectable marker to overcome inefficient endogenous homologous recombination. Elimination of the selectable marker and shortening the homology arms would greatly facilitate targeting vector construction, and would obviate the need to subsequently remove the selectable marker (usually leaving a recombinase recognition sequence as a residual scar). We asked if greater HDR efficiency achieved using Cas9 would permit a simple vector consisting of the desired knockin modification flanked by 50 base pair homology regions. We generated such a simplified dsDNA donor to introduce the FLAG-BIO epitope tag into the *Mef2c* C-terminus (Fig. 5B). The simplified targeting vector was co-transfected with pCas9-GFP and appropriate gRNA into mESCs. FACS-sorted, GFP^+^ cells were clonally expanded. Genotyping of DNA pooled from clones showed that there were two PCR bands in the Cas9 co-transfected group, with the larger amplicon arising from insertion of the FLAG-BIO tag at the *Mef2c* C-terminus (Fig. 5C). Genotyping of 22 individual clones showed that 8 (36.3%) contained the Flagbio insertion. We validated the function of the inserted FLAG-BIO tag by transfecting two genotype positive clones with a BirA expression plasmid. BirA recognizes the BIO peptide and biotinylates it [15]. To test the successful tagging and biotinylation of *Mef2c*, ESC lysates were incubated with streptavidin beads, and material bound to the beads was immunoblotted with anti-FLAG antibody. This showed that a FLAG-tagged protein with appropriate molecular weight for MEF2C was efficiently pulled down on the SA beads (Fig. 5D), confirming the functionality of the knockin allele.

A 99 bp, the FLAG-BIO epitope tag is still relatively small. To investigate the ability of Cas9 and a simplified targeting vector containing only 50 bp homology arms to knockin a larger foreign DNA fragment, we attempted to fuse GFP to MEF2C. This targeting vector contained the GFP open reading frame (720 bp) flanked by the same 50 bp homology arms as used for introduction of FLAG-BIO (Fig. 5E). We co-transfected this targeting vector with pCas9-GFP and *Mef2c* gRNA into mESCs. GFP^+^ cells were isolated by FACS and clonally expanded. Pooled DNA from resultant clones was positive for a diagnostic PCR product (Fig. 5F). Genotyping 46 individual clones identified 2 positive clones (4.3%). Embryoid body differentiation of one of the positive clones induced GFP fluorescence in regions of the embryoid body, suggesting that the GFP tag was functional (Fig. 5G). Sequencing of RTPCR product confirmed the desired fusion of GFP cDNA to *Mef2c*. These data show that HDR driven by Cas9 can be used for targeted insertion of a fragment several hundreds of base pairs long using a simple targeting vector with only 50 bp homology arms and no selectable marker.

**Figure 6:**
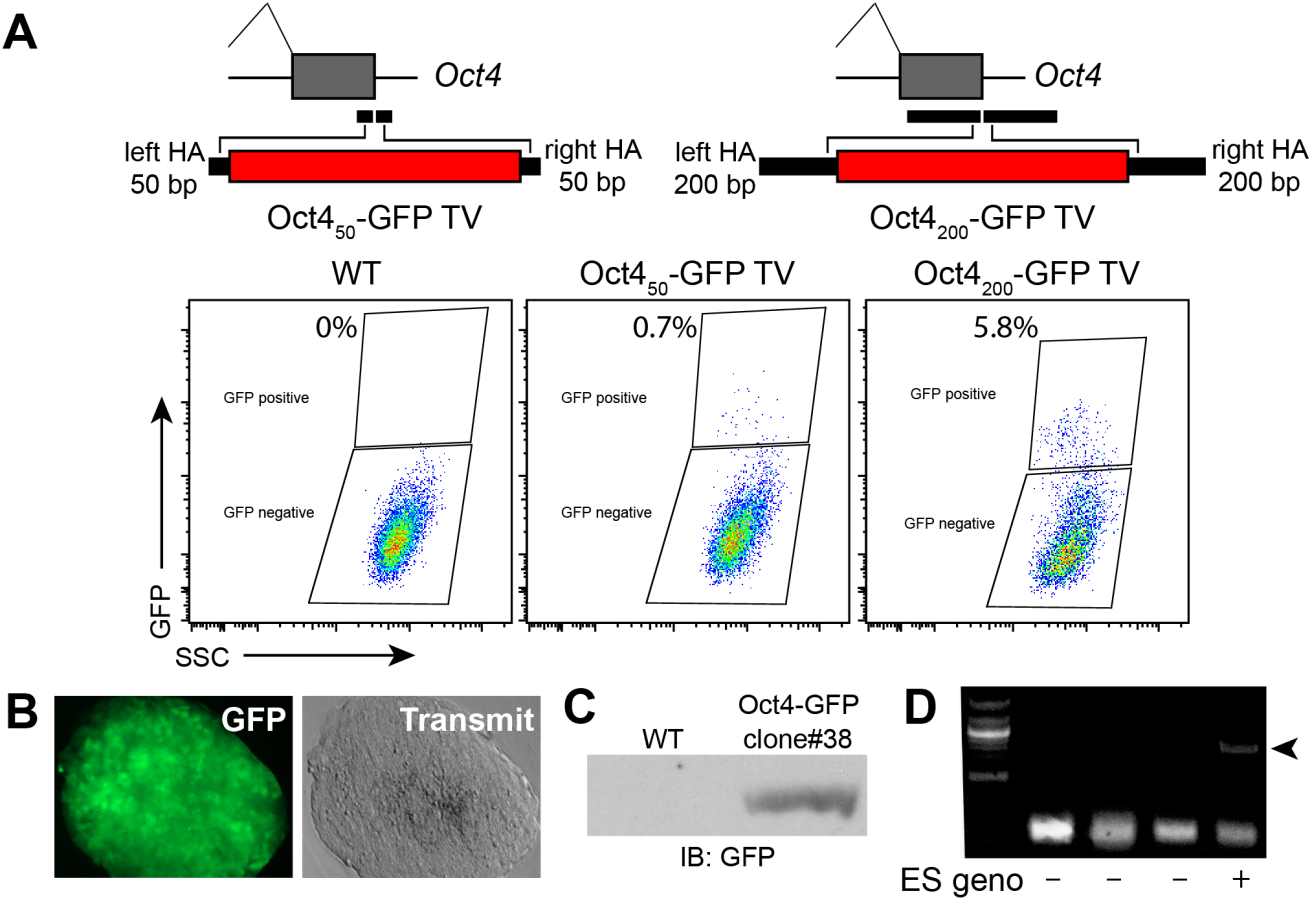
Effect of homology arm length for Cas9-mediated knockin. **A.** GFP knockin at Oct4 C-terminus using 50 bp or 200 bp homology arms. Knockin efficiency was measured by FACS for GFP expression. **B–C.** GFP expression in GFP^+^ FACS clones by microscopy and western blotting. **D.** PCR genotyping of individual ESC clones. The PCR product (arrowhead) is diagnostic of knockin at *Oct4*.

To see if another locus can be effectively modified by this strategy, we targeted *Oct4*, a transcription factor expressed in mESCs. Oct4_50_-GFP donor, containing 50 bp homology arms, gRNA and Cas9 were co-transfected into mESCs. In this case, it was not possible to enrich for transfected cells by FACS. After 10 days of ESC culture, FACS analysis showed that this treatment generated 0.7% GFP^+^ cells (Fig. 6A). Since this experiment did not enrich for transfected cells, the frequency of desired targeting amongst transfected cells is likely more then 5-fold higher (assuming a transfection efficiency of 20%). Genotype positive clone #38 stably expressed OCT4-GFP, as confirmed by fluorescent imaging and western blotting (Fig. 6B–C).

To determine if longer homology arms could increase efficiency of HR-mediated knockin, we tested 200 bp homology arms, as arms of this length can easily be synthesized as oligonucleotides. Using Gibson assembly, we generated an Oct4_200_-GFP donor, containing 200 bp homology arms. We introduced this construct into mESCs with Cas9 and appropriate gRNA. After 10 days of ESC culture, FACS analysis showed that this treatment generated 5.8% GFP^+^ cells (Fig. 6A). Genotyping of GFP^+^ clones confirmed appropriate knockin at the *Oct4* locus (Fig. 6D). These data show that increasing homology arm length from 50 to 200 bp increased targeting efficiency by more than 9-fold.

## Discussion

Scarless, targeted genome modification using designer site-directed nucleases such as CRISPR/Cas9 is a game changing technology. Here we optimized several aspects of this approach to enhance its efficiency. In a system where there is no selection for the desired product, the most important step is to achieve efficient transfection and then to enrich for transfected cells. Nucleofection and LF3000 are good transfection options for murine ESCs. Enrichment for transfected cells can be achieved by FACS sorting for GFP co-expressed from the hCas9-GFP plasmid, or co-transfection with a puromycin resistance marker followed by transient puromycin selection [11]. We found that both enrichment strategies were effective, although GFP FACS selection may yield a higher fraction of mutants with targeting of both alleles. On the other hand, one advantange of transient puromyocin selection is that cells do not need to leave the controlled environment of the cell culture room and be processed on a FACS machine, with attendant risks of mycoplasma or bacterial contamination.

It is important to consider downstream genotyping when designing a gene targeting strategy. Small indel mutations introduced by NHEJ using a single gRNA can be difficult to detect without DNA sequencing. Sanger sequencing can become expensive when many clones need to be screened. Moreover standard Sanger sequencing data can be difficult to interpret because indel mutations cause chromatogram phase shifts, so that it can be difficult to distinguish homozygous and heterozygous mutants [16]. We showed that dual gRNAs do not reduce (and may marginally enhance) targeting efficiency and simplify genotyping by causing more extensive deletions that are easily detected by PCR genotyping. This strategy can also be used to excise regions of DNA, such as transcriptional regulatory sequences. One downside to the use of paired gRNAs for gene knockout is that use of two gRNAs might increase the chance of off-target genome modification. We showed that one gRNA plus a DNA donor containing a stop codon enhances functional gene knockout. Addition or removal of restriction sites in addition to a stop codon or frameshift mutation in an HDR donor is an alternative strategy to facilitate downstream genotyping [17], although HR-dependent targeting tends to be several fold less efficient than NHEJ.

It has been suggested that a “dual nick” strategy, in which two gRNAs direct the Cas9D10A single strand nickase activity to nearby sites to yield an effective double strand break, will be a effective means to minimize off-target mutagenesis [11], [13], because the two gRNAs will rarely yield nearby nicks outside of the target locus. Our data support this idea, since mutagenesis by the nickase plus a single gRNA occurred at a low (but detectable) frequency. Paired gRNAs that induced nearby nicks stimulated mutagenesis by approximately 20-fold. Thus, efficient mutagenesis with minimal off-target activity could best be achieved using the Cas9D10A nickase and paired gRNAs. However, genotyping of the resulting small indel mutations will be more labor intensive and costly.

CRISPR/Cas9 greatly facilitates targeted genome modification and introduction of new DNA sequences at a target locus. This technology will facilitate generation of novel reagents, such as proteins labeled with affinity or fluorescent tags. We explored the variables that influence efficient introduction of new DNA sequences at a target locus. The size of the insert was an important variable, with larger inserts reducing the efficiency of genome modification. For instance, at the *Mef2c* locus, changing the insert length from 99 bp to 720 bp reduced modification frequency 9-fold (36.3% to 4.3%). On the other hand, increasing the homology arm size increased modification frequency: at the Oct4 locus, increasing the homology arm size from 50 bp to 200 bp increased the frequency of inserting a 720 bp fragment by 8-fold (0.7% to 5.8%; note these are the frequencies of modification of all cells, not transfected cells). Using TALENs to induce HDR modification of a single nucleotide, Hendel et al. showed that 400 bp homology arms improved HDR about 3-fold compared to 200 bp, while 800 bp was equivalent to 400 bp [18].

Increasing homology arm size can compensate for increased insert size. For example, at the Mef2c locus, an 2135 bp fragment was introduced with 19.6% efficiency with 1 kb and 4 kb homology arms. Using Gibson assembly [8], 200–400 bp homology arms can be added to an insert with ease. Therefore, we recommend use of homology arms of this length for larger DNA insertions. For shorter inserts (∼100 bp), 50 bp homology arms are sufficient, so that the entire targeting construct can be synthesized as an oligonucleotide.
